# Impact of downtime on surgical outcomes after repair of acute type A aortic dissection presenting with cardiopulmonary arrest

**DOI:** 10.1016/j.xjse.2026.100133

**Published:** 2026-06-12

**Authors:** So Izumi, Fuyuko Miki, Reiko Kanno, Takanori Tsujimoto, Chikashi Nakai, Kazumasa Edono, Ryouichi Mizoue, Shinichi Ijuin, Satoshi Ishihara, Takuro Tsukube

**Affiliations:** a Division of Cardiovascular Surgery, Japanese Red Cross Kobe Hospital & Hyogo Emergency Medical Center, Kobe, Japan; b Department of Clinical Engineering, Japanese Red Cross Kobe Hospital & Hyogo Emergency Medical Center, Kobe, Japan; c Department of Anesthesiology, Japanese Red Cross Kobe Hospital, Kobe, Japan; d Department of Emergency & Critical Care Medicine, Hyogo Emergency Medical Center, Kobe, Japan

**Keywords:** cardiopulmonary arrest, acute type A aortic dissection, cardiopulmonary resuscitation, downtime, immediate aortic repair

## Abstract

**Objective:**

The management of acute type A aortic dissection with cardiopulmonary arrest remains controversial. We evaluated the impact of downtime on outcomes after aortic repair in this high-risk population.

**Methods:**

We retrospectively reviewed 711 patients with acute type A aortic dissection admitted between August 2003 and December 2024. Patients were categorized according to the presence of cardiopulmonary arrest and whether immediate aortic repair was performed. Among surgically treated patients with cardiopulmonary arrest, downtime was defined as the interval from collapse to return of spontaneous circulation or establishment of extracorporeal cardiopulmonary resuscitation. Early mortality, long-term survival, and discharge destination were analyzed.

**Results:**

Among 222 patients with cardiopulmonary arrest, 30 underwent aortic repair and 192 did not. Aortic repair was performed in 489 patients without cardiopulmonary arrest. Early mortality was 100% in the cardiopulmonary arrest without surgery group, 40.0% in the patients with surgery group, and 5.1% in the noncardiopulmonary arrest group (*P* < .001). In the surgically treated cardiopulmonary arrest group, 6 patients were discharged home, and the 5-year survival rate was 30.5%. In the multivariable analysis, coronary malperfusion (*P* = .002) and longer downtime (*P* = .009) were associated with increased late mortality. Downtime predicted discharge home with an area under the curve of 0.892, and the exploratory optimal cutoff was 18 minutes.

**Conclusions:**

Acute type A aortic dissection presenting with cardiopulmonary arrest carries exceptionally high risk. However, discharge home and long-term survival may be achieved in selected patients when downtime is short and immediate aortic repair follows successful resuscitation.

**Figure:**
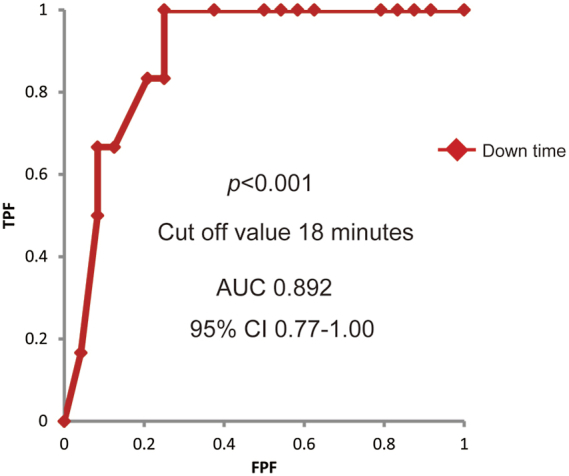
Shorter downtime predicted for returning home, with an optimal threshold of 18 minutes.

**Central Message**

Although acute type A aortic dissection with cardiopulmonary arrest is associated with poor prognosis, discharge home can be achieved when downtime is short and aortic repair is performed immediately.

**Perspective**

Performing aortic repair in patients with acute type A aortic dissection accompanied by cardiopulmonary arrest remains controversial. When resuscitation is achieved within a short timeframe, immediate aortic repair could be justified in selected patients because meaningful recovery and long-term survival remain achievable.

See Commentary on page XXX.

Preoperative cardiopulmonary arrest (CPA) is the most catastrophic presentation of acute type A aortic dissection (ATAAD) and remains associated with extremely poor outcomes. Coronary malperfusion is another life-threatening complication of ATAAD and can precipitate malignant arrhythmias and CPA.^1^^,^^2^ Recent improvements in emergency medical systems and the growing use of extracorporeal cardiopulmonary resuscitation (ECPR) have increased the number of patients with ATAAD who reach hospital after CPA.^1^^,^^3^

In parallel, outcomes after ATAAD repair have improved substantially, with contemporary nationwide data from Japan reporting hospital mortality less than 10%.^4^ However, the prognosis for ATAAD complicated by CPA remains dismal.^1^^,^^2^^,^^5^ Indications and optimal timing for definitive aortic repair after CPA are still debated, particularly for patients requiring prolonged resuscitation or ECPR.

The clinical impact of resuscitation duration can be framed by “downtime,” defined as the interval from collapse to return of spontaneous circulation (ROSC) or, when ROSC is not achieved, to establishment of ECPR. Although downtime is biologically linked to neurologic injury and multiorgan ischemia, its relationship to long-term survival and meaningful recovery after ATAAD repair has not been well characterized. We therefore analyzed a 2-decade, single-center experience to evaluate early and late outcomes after ATAAD repair in patients presenting with CPA, identify predictors of mortality, and determine a clinically useful downtime threshold associated with discharge to home.

## Methods

### Study Population and Ethics

We retrospectively reviewed all patients diagnosed with ATAAD who were transported to the Japanese Red Cross Kobe Hospital and Hyogo Emergency Medical Center between August 2003 and December 2024. The institutional Committee for the Protection of Human Subjects approved data collection and analysis (no. 411; December 25, 2024) and waived the requirement for individual informed consent. Follow-up information was obtained by chart review and telephone contact with the patient, a family member, or the primary physician.

### Definitions

Cardiac arrest was defined as sudden cessation of cardiac activity with unresponsiveness, absent normal breathing, and no signs of circulation.^6^ ROSC was defined as a palpable pulse with systolic blood pressure ≥60 mm Hg without ongoing cardiopulmonary resuscitation (CPR). Downtime was defined as the interval from collapse to ROSC or, when ROSC was not achieved, from collapse to establishment of ECPR. Time from onset to operating room was defined as the time from onset of symptom to arrival at operation room.

### Resuscitation Strategy and ECPR Criteria

All patients with CPA underwent immediate conventional CPR. Patients without ROSC who met institutional criteria underwent ECPR (venoarterial extracorporeal membrane oxygenation). Eligibility criteria were (1) collapse-to-hospital arrival <45 minutes; (2) aged 15 to 75 years; and (3) an initial rhythm of ventricular fibrillation/ventricular tachycardia or pulseless electrical activity at CPA onset.^7^ ECPR was generally initiated when at least 3 criteria were met. During CPR, a 17-Fr arterial cannula and 21-Fr venous cannula were inserted via the femoral artery and vein, respectively, and extracorporeal membrane oxygenation was initiated with a target flow of approximately 3 L/min. Controlled pericardial drainage was performed when significant pericardial effusion with tamponade physiology was suspected.^8^ ATAAD was generally diagnosed by contrast-enhanced computed tomography (CT). For patients who experienced out-of-hospital cardiac arrest (OHCA), CT was performed after ROSC when feasible. The decision to proceed with immediate aortic repair after CPA was generally made when ROSC was achieved or ECPR was established, ATAAD was confirmed by contrast-enhanced CT, and no obvious intracranial hemorrhage or severe hypoxic encephalopathy was identified on neurologic assessment and head CT when feasible.

### Operative Technique

Aortic repair was performed using cardiopulmonary bypass and moderate hypothermic circulatory arrest (target rectal temperature 25 °C) with antegrade selective cerebral perfusion via the left subclavian, left common carotid, and innominate arteries. In hemodynamically unstable patients with CPA, arterial cannulation was generally performed through the femoral artery to establish cardiopulmonary bypass rapidly. Total arch replacement with an elephant trunk was performed when the intimal tear involved the distal transverse arch. Hemiarch replacement was performed when the tear was confined to the proximal arch, using antegrade or retrograde cerebral perfusion.

### Outcomes

Early outcome was assessed using 30-day mortality. Long-term outcome was assessed using 5-year survival. Neurologic status was evaluated using the Glasgow Coma Scale in the preoperative and early postoperative periods. Severe consciousness disorder was defined as lower than 8 on the Glasgow Coma Scale; transient disorders were not counted as events.^9^ Functional recovery was assessed by discharge destination; discharge to home was used as a pragmatic marker of meaningful recovery.

### Statistical Analysis

Categorical variables are presented as number (percentage) and continuous variables as median (interquartile range). For between-group comparisons we used χ^2^ testing for proportions. Univariable logistic regression identified predictors of early mortality in the CPA group. Long-term survival was estimated using Kaplan-Meier analysis and compared using the log-rank test. Multivariable Cox proportional hazards models assessed predictors of late mortality. Receiver operating characteristic analysis was used to identify a downtime threshold associated with discharge to home. Analyses were performed using Bell Curve for Excel (version 4.00; Social Survey Research Information).

## Results

During the study period, 711 patients with ATAAD were transported to our center; 197 presented with OHCA and 25 experienced in-hospital CPA, for a total of 222 CPA presentations. Among all 4147 patients with OHCA transported to our center, ATAAD accounted for 4.8% (197/4147).

**Figure 1:**
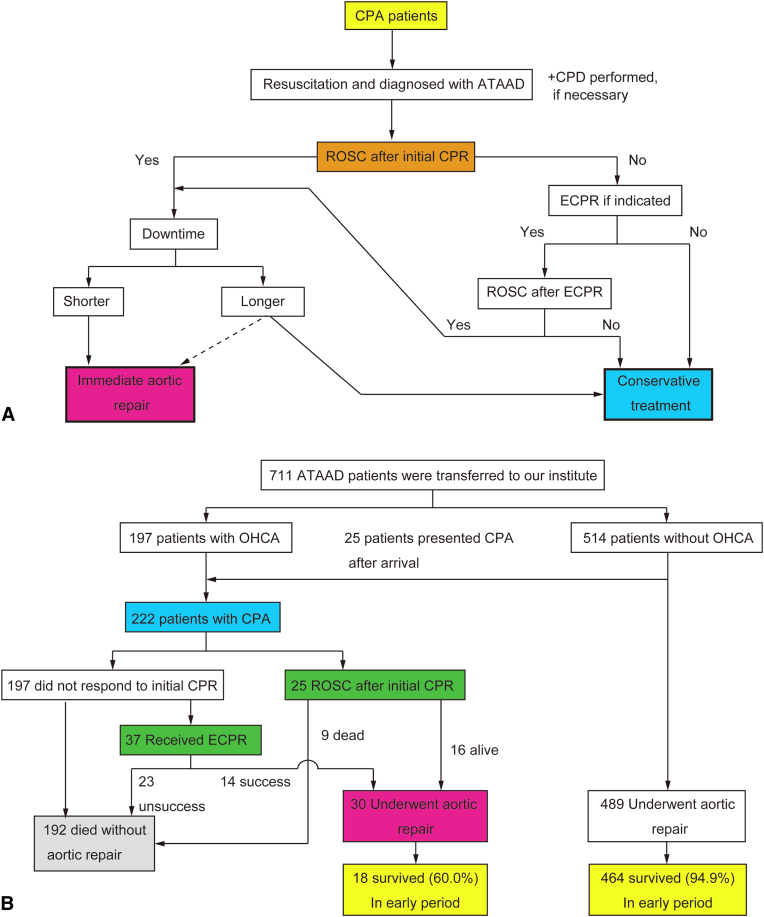
A and B, Current therapeutic strategy and enrollment and early outcomes. *CPA*, Cardiopulmonary arrest; *ATAAD*, acute type A aortic dissection; *CPD*, controlled pericardial drainage; *ROSC*, return of spontaneous circulation; *CPR*, cardiopulmonary resuscitation; *ECPR*, extracorporeal cardiopulmonary resuscitation; *OHCA*, out-of-hospital cardiac arrest.

The presumed mechanisms of CPA were cardiac tamponade in 53.1% (n = 118), aortic rupture in 9.0% (n = 20), coronary malperfusion in 9.9% (n = 22), and other or unknown causes in 27.9% (n = 62). All patients with CPA were managed according to an institutional pathway (Figure 1, *A*). Because cardiac tamponade was frequently observed, controlled pericardial drainage was applied in many cases. ROSC after initial CPR was achieved in 25 patients; 16 proceeded directly to aortic repair. Among 197 patients without ROSC after initial CPR, 37 underwent ECPR. Among the 37 patients who underwent ECPR, the initial documented rhythm was pulseless electrical activity in 28, ventricular tachycardia/ventricular fibrillation in 5, and unknown in 4. The 4 patients with unknown rhythm were treated during the early phase of ECPR introduction; under the current institutional protocol, ECPR is considered only for patients with pulseless electrical activity or ventricular tachycardia/ventricular fibrillation. Of those, 14 underwent aortic repair and 23 died despite receiving ECPR (Figure 1, *B*). Eventually, 30 patients underwent aortic repair after CPA. In the same time period, 489 patients with ATAAD underwent aortic repair without preoperative CPA.

Patients were categorized according to the presence of CPA and whether immediate aortic repair was performed. The 222 patients with CPA were defined as the full CPA cohort. Among them, 30 patients who underwent aortic repair were defined as the CPA with surgery group, and 192 patients who did not undergo aortic repair were defined as the CPA without surgery group. The 489 patients who underwent aortic repair without preoperative CPA were defined as the non-CPA with surgery group. Baseline characteristics are summarized in Table 1, Table 2, Table 3, and Table E1. Patients in the CPA without surgery group had the most severe preoperative condition, and all died soon after admission. Downtime was available for all patients in the CPA with surgery group but was missing in 13 of 192 patients (6.8%) in the CPA without surgery group.

**Table 1:** Comparisons of baseline characteristics between full CPA and non-CPA groups

Characteristic	Full CPA (n = 222)	Non-CPA (n = 489)	*P*
Age, y	75 (68-83)	73 (63-81)	<.001
Male	103 (46.4)	224 (45.8)	.88
Shock	222 (100.0)	91 (18.6)	<.001
Controlled pericardial drainage	47 (21.1)	54 (11.0)	<.001
Preoperative Glasgow Coma Scale	3 (3-3)	15 (15-15)	<.001
Early mortality	204 (91.9)	25 (5.1)	<.001
Cumulative 5-y survival rate, %	4.1	80.1	<.001

*CPA*, Cardiopulmonary arrest.

**Table 2:** Comparisons of baseline characteristics between CPA with surgery and non-CPA with surgery groups

Characteristic	CPA with surgery (n = 30)	Non-CPA with surgery (n = 489)	*P*
Age, y	74 (64-80)	73 (63-81)	<.001
Male	15 (50.0)	224 (45.8)	.99
Shock	30 (100.0)	91 (18.6)	<.001
Controlled pericardial drainage	19 (63.3)	54 (11.0)	<.001
Preoperative Glasgow Coma Scale	4 (3-6)	15 (15-15)	<.001
Time from onset to OR, min	212 (178-336)	304 (223-511)	.006
Malperfusion	24 (80.0)	139 (28.4)	<.001
Cerebral	16 (53.3)	72 (14.8)	<.001
Coronary	9 (30.0)	8 (1.6)	<.001
Kidney	1 (3.3)	23 (4.7)	1.00
Range of replacement			
Hemiarch	19 (63.3)	280 (57.3)	.51
Partial arch	3 (10.0)	41 (8.4)	.73
Total arch	8 (26.7)	168 (34.4)	.39
Concomitant CABG	9 (30.0)	10 (2.0)	<.001
Operation time, min	498 (425-607)	429 (361-536)	<.001
Cardiopulmonary bypass time, min	270 (244-327)	241 (197-296)	.008
Myocardial ischemic time, min	191 (149-217)	154 (123-193)	.01
Cerebral perfusion time, min	75 (49-107)	78 (50-146)	.27
Early mortality	12 (40.0)	25 (5.1)	<.001
Re-thoracotomy for bleeding	11 (36.7)	47 (9.6)	<.001
New onset of stroke	12 (40.0)	60 (12.3)	<.001
Postoperative severe consciousness disorder	19 (63.3)	40 (8.2)	<.001
Cumulative 5-y survival rate, %	30.5	80.1	<.001

*CPA*, Cardiopulmonary arrest; *OR*, operating room; *CABG*, coronary artery bypass grafting.

**Table 3:** Comparisons of baseline characteristics between CPA without surgery and CPA with surgery groups

Characteristic	CPA without surgery (n = 192)	CPA with surgery (n = 30)	*P*
Age, y	77 (68-83)	74 (64-80)	.03
Male	88 (45.8)	15 (50.0)	.67
Controlled pericardial drainage	28 (14.5)	19 (63.3)	<.001
Preoperative Glasgow Coma Scale	3 (3-3)	4 (3-6)	<.001
OHCA	181 (94.3)	16 (53.3)	<.001
Initial documented rhythm			
PEA	100 (52.1)	28 (93.3)	<.001
Asystole	67 (34.9)	0	<.001
VT/VF	4 (2.1)	2 (6.7)	.19
Unknown	21 (10.9)	0	.09
Cause of CPA			
Cardiac tamponade	97 (50.5)	21 (70.0)	.04
Coronary malperfusion	13 (6.8)	9 (30.0)	<.001
Aortic rupture	20 (10.4)	0	.08
Unknown	62 (27.9)	0	<.001
ROSC after initial CPR	9 (4.7)	16 (53.3)	<.001
ECMO initiated	23 (17.6)	14 (46.7)	<.001
Downtime	37 (32-45)	21 (12-30)	<.001
Early mortality	192 (100.0)	12 (40.0)	<.001

Categorical variables are presented as number (percentage) and continuous variables as median (interquartile range). *CPA*, Cardiopulmonary arrest; *OHCA*, out-of-hospital cardiopulmonary arrest; *PEA*, pulseless electrical activity; *VT*, ventricular tachycardia; *VF*, ventricular fibrillation; *ROSC*, return of spontaneous circulation; *CPR*, cardiopulmonary resuscitation; *ECMO*, extracorporeal membrane oxygenation.

**Figure 2:**
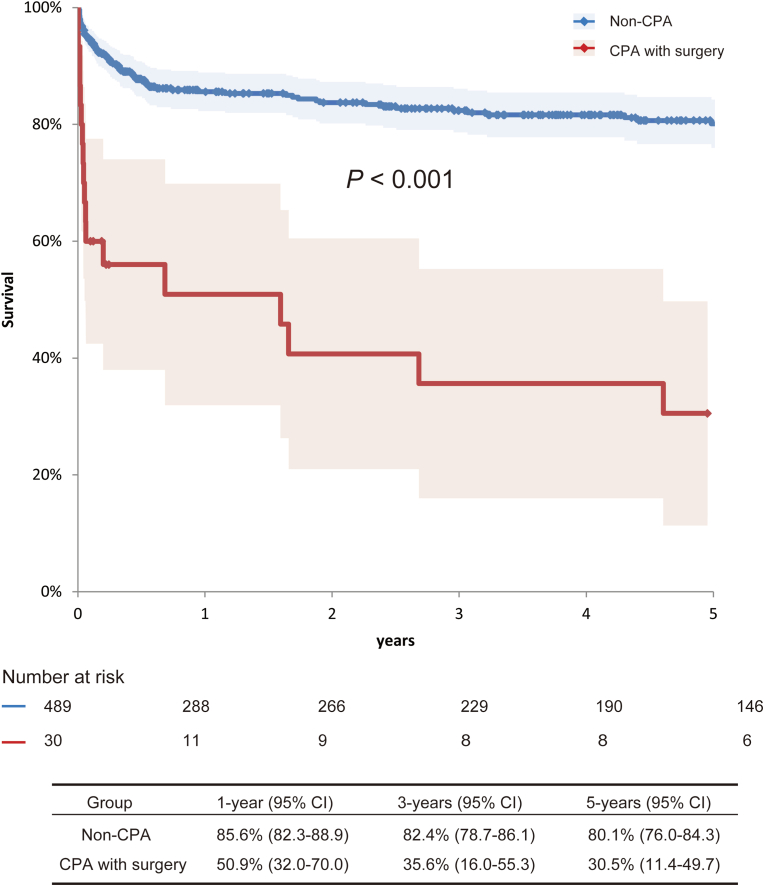
Kaplan-Meier survival curves of the CPA with surgery group and the non-CPA with surgery group, 95% CI. *CPA*, Cardiopulmonary arrest.

Early mortality was significantly greater in the CPA with surgery group than in the non-CPA with surgery group (40.0% vs 5.1%, *P* < .001), and long-term survival was lower (5-year survival 30.5% vs 80.1%, *P* < .001; Figure 2). Univariable logistic regression analysis showed that younger age, male sex, and coronary malperfusion were associated with early mortality in the CPA with surgery group (Table 4).

**Table 4:** Univariable logistic regression analysis of early mortality in CPA with surgery

Risk factors	OR	95% CI	*P*
Age	0.93	0.87-0.99	.04
Male	6.00	1.17-30.7	.03
OHCA	2.50	0.54-11.4	.23
ECPR	4.00	0.85-18.8	.08
Downtime	1.07	0.99-1.16	.08
Time from ROSC to operating room	1.00	0.99-1.00	.93
Coronary malperfusion	11.2	1.73-72.3	.01

*CPA*, Cardiopulmonary arrest; *OR*, odds ratio; *OHCA*, out-of-hospital cardiac arrest; *ECPR*, extracorporeal cardiopulmonary resuscitation; *ROSC*, return of spontaneous circulation.

Among surgically treated patients with CPA, early mortality was 57.1% in the ECPR group and 25.0% in the ROSC-without-ECPR group. Six patients who received ECPR survived beyond 30 days, but only 1 was discharged home; this patient survived for 2 years. The incidence of postoperative severe consciousness disorder did not differ significantly between the groups. These subgroup data are summarized in Table E2.

Eighteen patients with CPA survived beyond 30 days; 6 were discharged home. The mean downtime of the 6 survivors was 6.6 minutes. Five of the 6 patients were resuscitated with initial CPR, and ECPR was initiated in only 1 patient. The presumed mechanism of CPA was cardiac tamponade in all 6 patients, and controlled pericardial drainage was performed before surgery in all 6 patients. The other 12 patients with CPA survived longer than 30 days, but none were discharged home. The mean downtime of these 12 patients was 21.3 minutes. ECPR was initiated in 5 patients. Two patients died in our institute as the result of multiple organ failure and multiple cerebral infarctions. The remaining 10 patients were transferred to rehabilitation hospitals with severe deterioration in activities of daily living, and the final status was not followed. In the Cox model, longer downtime and coronary malperfusion were independently associated with greater late mortality (hazard ratio, 1.06 per minute; 95% CI 1.01-1.11, *P* = .009; and hazard ratio, 5.63; 95% CI, 1.86-17.0, *P* = .002; Table 5).

**Table 5:** Multivariable predictors of long-term survival

Risk factors	HR	95% CI	*P*
Downtime	1.06	1.01-1.11	.009
Coronary malperfusion	5.63	1.86-17.0	.002

*HR*, Hazard ratio.

**Figure 3:**
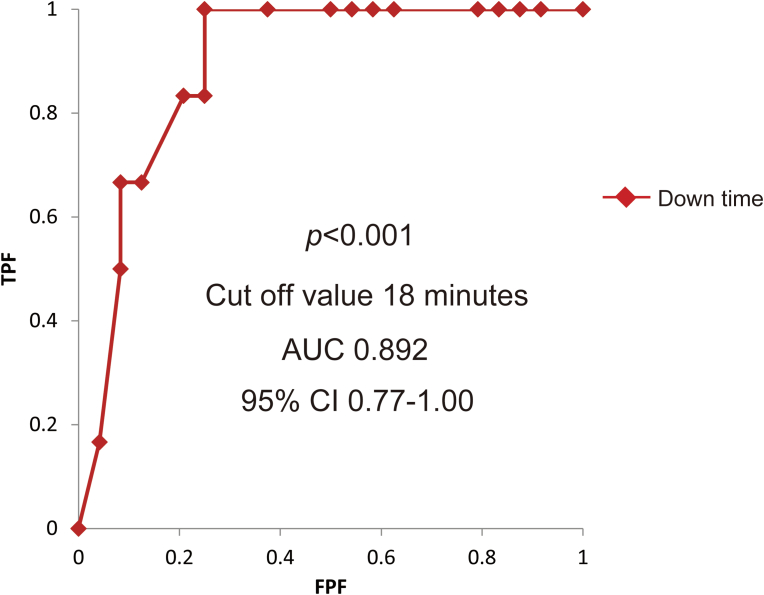
ROC curve for downtime predicting discharge home. *ROC*, Receiver operating characteristic; *TPF*, true-positive fraction; *FPF*, false-positive fraction.

Downtime was closely related to neurologic recovery and discharge destination (Table 6). Although some patients with downtime >20 minutes survived beyond 30 days, severe consciousness disorder was common and no patient with downtime ≥21 minutes was discharged home. Downtime showed good discrimination for predicting discharge to home (area under the curve, 0.892; 95% CI, 0.77-1.00, *P* < .001), with an optimal threshold of 18 minutes (Figure 3).

**Table 6:** Downtime and parameters of surgical results of the patient with ROSC

Characteristic	Downtime, min			
	≤10 (n = 8)	11-20 (n = 7)	21-30 (n = 10)	31-40 (n = 5)
30-d mortality, n (%)	1 (12.5)	2 (28.6)	4 (40.0)	2 (40.0)
Consciousness disorder, n (%)	2 (25.0)	4 (57.1)	10 (100.0)	4 (80.0)
Discharge to home, n (%)	5 (62.5)	2 (28.5)	0	0
5-y survival, %	43.8	53.5	0	0

*ROSC*, Return of spontaneous circulation.

## Discussion

This study shows that ATAAD presenting with CPA is associated with markedly worse early and late outcomes after repair than ATAAD without CPA. Nevertheless, meaningful recovery—reflected by discharge to home—was achievable in a subset of patients. Downtime and coronary malperfusion were most strongly associated with long-term outcome.

Previous series report that 3.8% to 13.1% of patients with ATAAD undergoing repair experience preoperative CPA,^5^^,^^10^^,^^11^ and ATAAD accounts for a clinically important proportion of OHCA.^12^ The proportion of patients presenting with CPA may have been greater than in previous surgical series because our cohort included all patients with ATAAD transported to our emergency center, including patients with unwitnessed out-of-hospital cardiac arrest who were diagnosed with ATAAD only after arrival.^2^^,^^5^^,^^10^^,^^11^ Pan and colleagues^10^ reported that midterm outcomes after surviving the initial period were acceptable and concluded that preoperative cardiac arrest should not be considered an absolute contraindication for aortic repair. Our findings are consistent with these data, while further emphasizing that favorable long-term outcomes are possible when resuscitation is rapid and definitive repair is not delayed. The shorter time from symptom onset to the operating room in the CPA with surgery group likely reflected the greater clinical urgency and direct transfer to the operating room after ROSC or ECPR establishment.

Mechanisms of CPA in ATAAD commonly include tamponade, aortic rupture, and coronary malperfusion. In our cohort, aortic rupture-associated CPA was uniformly fatal before repair, consistent with the challenge of achieving ROSC or establishing ECPR in the setting of ongoing exsanguination. By contrast, repair was feasible when tamponade or coronary malperfusion predominated. Controlled pericardial drainage may restore perfusion in critical tamponade and is supported by previous reports and contemporary expert guidance.^2^^,^^13^

Coronary malperfusion was associated with early mortality in our CPA cohort. PCI was attempted in only 2 patients with coronary artery dissection; both procedures were time-consuming, and both patients died after aortic repair. Because this experience was limited to 2 patients, it should be interpreted as contextual rather than definitive evidence against preoperative PCI. Although preoperative PCI has been proposed,^14^ it may delay central aortic repair and could worsen ischemia in other territories. A streamlined strategy that minimizes delay while addressing coronary compromise, such as expeditious repair with selective coronary artery bypass grafting, may be critical, but optimal pathways require further study.

Among the 23 patients with ECPR who did not undergo repair, the time from collapse to ECPR establishment ranged from 24 to 67 minutes. Despite ECPR initiation, definitive repair could not be performed because of persistent circulatory collapse, suspected or confirmed rupture, or severe hypoxic brain injury.^1^ In our ECPR protocol, the upper age limit was 75 years, which may be relatively high compared with protocols used in some other countries. Although the SAVE-J II study in Japan did not set an upper age limit,^15^ it reported a strong association between older age and poor prognosis. It appears that the criteria for ECPR, including age, remain a matter for debate. Therefore, our data do not justify a categorical recommendation against ECPR, but they suggest that meaningful recovery after ECPR is uncommon and should be interpreted in the context of patient selection, downtime, and the mechanism of arrest.

Downtime emerged as a key determinant of long-term outcomes. Uehara and colleagues^11^ reported that the duration of CPR was significantly shorter in survivors than in nonsurvivors (10 minutes vs 16.5 minutes), and all survivors who did not display neurologic deficits showed ROSC after a pericardiotomy. They suggested that CPR lasting more than 15 minutes could be a contraindication for ATAAD repair. Because our study included patients who were able to live independently despite some residual neurologic deficits, the downtime might be slightly longer than in this study. Accordingly, for patients requiring prolonged resuscitation, proceeding to emergent repair should be individualized, incorporating downtime, suspected mechanism (tamponade vs aortic rupture), neurologic examination when available, and the feasibility of rapid definitive repair.

This study has limitations inherent to its retrospective single-center design and the small number of surgically treated patients with CPA. Because aortic repair was only possible in cases in which the conditions were suitable, selection bias might be affecting the results. Therefore, the identified downtime threshold applies only to surgically treated patients and should not be interpreted as a universal cutoff for proceeding with repair. The ROC-derived 18-minute cutoff should be considered exploratory because it was derived from a small operative cohort with only 6 patients discharged home. Detailed long-term neurologic outcomes were not consistently available, especially after transfer to other hospitals. Therefore, discharge to home and Glasgow Coma Scale were used as pragmatic, but imperfect, surrogate markers of meaningful neurologic and functional recovery. An exploratory era analysis showed no significant difference in 30-day mortality between the first and second decades among all patients with CPA or among surgically treated CPA patients. However, this analysis was underpowered, and temporal changes in prehospital care, transport systems, ECPR practice, cerebral protection, perioperative management, and surgical experience may have influenced patient selection and outcomes. For patients who experienced OHCA, the exact interval from collapse to initiation of CPR and the quality of bystander CPR were not consistently available. Because the operative CPA cohort was small, no a priori power calculation was performed, and the regression analyses should be interpreted as exploratory and potentially underpowered. These limitations should be considered when applying the proposed downtime threshold across systems.

## Conclusions

ATAAD presenting with CPA carries exceptionally high risk after repair, but favorable long-term outcomes—including discharge to home—can be achieved in selected patients when downtime is short and immediate aortic repair follows successful resuscitation.

## Conflict of Interest Statement

The authors reported no conflicts of interest.

The *Journal* policy requires editors and reviewers to disclose conflicts of interest and to decline handling or reviewing manuscripts for which they may have a conflict of interest. The editors and reviewers of this article have no conflicts of interest.

## Figures and Tables

**Table E1:** Comparisons of baseline characteristics between CPA without surgery and non-CPA with surgery groups

Characteristic	CPA without surgery (n = 192)	Non-CPA with surgery (n = 489)	*P*
Age, y	77 (68-83)	73 (63-81)	<.001
Male	88 (45.8)	224 (45.8)	.99
Shock	192 (100.0)	91 (18.6)	<.001
Controlled pericardial drainage	28 (14.5)	54 (11.0)	.20
Preoperative GCS	3 (3-3)	15 (15-15)	<.001
Early mortality	192 (100.0)	25 (5.1)	<.001

Categorical variables are presented as number (percentage) and continuous variables as median (interquartile range). *CPA*, Cardiopulmonary arrest; *GCS*, Glasgow Coma Scale.

**Table E2:** Comparison of patients with and without ECPR in CPA with surgery group

Characteristic	ECPR (n = 14)	ROSC without ECPR (n = 16)	*P*
Age, y	64 (51-73)	80 (74-81)	<.001
Male	10 (71.4)	5 (31.3)	.02
Controlled pericardial drainage	5 (35.7)	15 (93.8)	.001
OHCA	7 (50.0)	9 (56.3)	.73
Initial documented rhythm			
PEA	12 (85.7)	16 (100.0)	.20
VT/VF	2 (14.3)	0	.20
Cause of CPA			
Cardiac tamponade	6 (42.9)	15 (93.4)	.004
Coronary malperfusion	8 (57.1)	1 (6.3)	.004
Downtime, min	28 (16-32)	19 (5-21)	.03
Time from onset to OR, min	216 (174-349)	228 (178-332)	.72
Cerebral malperfusion, n (%)	4 (28.6)	12 (75.0)	.01
Postoperative coma (GCS ≤8)	8 (57.1)	10 (62.5)	.77
Early mortality	8 (57.1)	4 (25.0)	.07
Discharge home	1 (7.1)	5 (31.2)	.17

Categorical variables are presented as number (percentage) and continuous variables as median (interquartile range). *ECPR*, Extracorporeal cardiopulmonary resuscitation; *CPA*, cardiopulmonary arrest; *ROSC*, return of spontaneous circulation; *OHCA*, out-of-hospital cardiopulmonary arrest; *PEA*, pulseless electrical activity; *VT*, ventricular tachycardia; *VF*, ventricular fibrillation; *GCS*, Glasgow Coma Scale; *OR*, operating room.
